# Phytochemical Targeting of Mitochondria for Breast Cancer Chemoprevention, Therapy, and Sensitization

**DOI:** 10.3390/ijms232214152

**Published:** 2022-11-16

**Authors:** Elizabeth R. M. Zunica, Christopher L. Axelrod, John P. Kirwan

**Affiliations:** Integrated Physiology and Molecular Medicine Laboratory, Pennington Biomedical Research Center, Baton Rouge, LA 70808, USA

**Keywords:** phytochemicals, mitochondria, breast cancer, chemoprevention, chemotherapy, chemosensitization

## Abstract

Breast cancer is a common and deadly disease that causes tremendous physical, emotional, and financial burden on patients and society. Early-stage breast cancer and less aggressive subtypes have promising prognosis for patients, but in aggressive subtypes, and as cancers progress, treatment options and responses diminish, dramatically decreasing survival. Plants are nutritionally rich and biologically diverse organisms containing thousands of metabolites, some of which have chemopreventive, therapeutic, and sensitizing properties, providing a rich source for drug discovery. In this study we review the current landscape of breast cancer with a central focus on the potential role of phytochemicals for treatment, management, and disease prevention. We discuss the relevance of phytochemical targeting of mitochondria for improved anti-breast cancer efficacy. We highlight current applications of phytochemicals and derivative structures that display anti-cancer properties and modulate cancer mitochondria, while describing future applicability and identifying areas of promise.

## 1. Introduction

Breast cancer is the most globally prevalent malignancy and a leading cause of cancer-related death. Advances in the diagnosis, classification, and treatment of breast cancer have dramatically evolved over the past 30 years, improving the survival and health-span of patients. Despite this, clinical outcomes remain highly dependent on the subtype, staging, and health of the individual, with worse survival in patients with later-stage and more aggressive breast cancers [1]. For example, aggressive, rapidly growing breast tumors are often initially responsive to first-line chemotherapies, but eventually recur and/or metastasize through death evasion and the onset of multi-drug resistance [2]. To address this issue, chemosensitizing agents are being developed to improve treatment response, reduce chemotherapy resistance, and ultimately increase survival [3]. Furthermore, patients with breast cancer concomitantly manage chronic diseases such as obesity, type 2 diabetes, arthritis, and hypertension, which may negatively affect treatment response, tolerability, and ultimately, survival [4,5]. Obesity is now considered an independent risk factor for the development, morbidity, recurrence, and mortality of breast cancer [6,7,8,9], and body mass index is used for more robust calculation of breast cancer risk [10] when considering use of chemoprevention drugs. Currently, medications for cancer prevention are limited to drugs that modulate estrogen signaling. Medications for weight-loss and diabetes management that target cancer-promoting processes are being investigated as potential chemoprevention agents in people identified with increased breast cancer risk [11]. Overall, there remains a grave need to identify complementary combinations of chemotherapies and chemosensitizers, as well as effective chemopreventive agents, for improved breast cancer survival.

Plants contain a diversity of compounds and metabolites with antineoplastic properties [12]. Advances in metabolite screening and bioinformatics have enabled more rapid discovery of lead drugs from natural products and secondary metabolites, such as phytochemicals [13]. Phytochemicals have been directly used as anti-cancer agents, as well as templates for the construction of synthetic antineoplastic agents, many of which are actively used for breast cancer treatment [14]. Deeper investigation of their mechanisms of action allows for improved combination use for the treatment of breast cancer. Additionally, phytochemicals such as curcumin and resveratrol display potential as chemosensitizers, compounds that improve chemotherapy response, and/or chemoprevention drugs, compounds that reduce a person’s risk for developing cancer or recurrence.

Chemosensitization strategies have mostly targeted cancer cell chemoresistance mechanisms, such as the ATP binding cassette (ABC) transporter proteins. Despite initial promise of first, second, and third generation chemosensitizers (approved drugs that are known to inhibit ABC transporters, modifications of these drugs to improve potency, and further modifications to reduce toxicity and retain activity, respectively), no compound has displayed the tolerability and efficacy requisite for use in patients. Given these recent failures and in an effort to reduce toxicity, natural products such as phytochemicals have gained attention to serve as frameworks for the development of fourth-generation compounds that are not toxic and offer unique structures to block ABC transporters and reverse multidrug resistance [15]. A number of phytochemicals that have been identified as functional components of natural anti-obesity and diabetes products [16] also demonstrate chemopreventive potential [17], indicating a conceivable mechanistic overlap in medically managing obesity and preventing cancer in people with a high risk of developing breast cancer.

Mitochondria are essential to cancer progression by providing the energy and macromolecules required to initiate and sustain growth, as well as modulating signaling processes that confer plasticity in changing environments [18]. Mitochondria are also positioned as the master-regulators of intrinsic apoptosis, and thus are commonly targeted in chemopreventive, chemotherapeutic, and chemosensitizing strategies. Mitocans are cancer therapeutics with known mitochondrial targets, which include popular and effective United States Food and Drug Administration (FDA) approved phytochemical-derived anti-cancer therapies, as well as novel phytochemicals with anti-cancer potential [19]. Phytochemical anti-cancer agents that target mitochondrial functions hold promise for the prevention of breast cancer and recurrence in women with higher risk of developing breast cancer. Additionally, they have been employed as effective chemotherapies for some breast cancers and show potential as chemosensitizers for improved treatment efficacy in advanced, aggressive cancers with low response to standard of care chemotherapies.

## 2. The Landscape of Breast Cancer

### 2.1. Epidemiology

Breast cancer is the most globally common malignancy, with an estimated 2.3 million new cases reported in 2020 [20,21]. Breast cancer accounts for 11.7% of all cancer cases and ~25% of cancer cases in women [21]. In the United States, 1 in 8 females will develop breast cancer during their lifetime, with over 275,000 cases annually diagnosed [1]. In the U.S., a modest decline in breast cancer incidence was observed in the early 2000s, plateauing for ~7 years, but more recently has shown a slight but steady rise [1]. Advances in early detection and treatment regimens have improved clinical outcomes for patients, depending on breast cancer subtype and disease risk factors [1]. Approximately 5–10% of breast cancers are hereditary [22], with the remaining 90–95% of cases influenced by phenotypic traits, environment, and lifestyle [23,24], indicating that primary risk factors associated with breast carcinogenesis are potentially modifiable.

The classification and diagnosis of breast cancer has evolved over time. Currently, the presence of hormone and growth factor receptors is used to determine tumor subtypes. The hormone receptor positive, human epidermal growth factor receptor 2 negative (HR+/HER2-; molecular subtype luminal A) subtype is the least aggressive and most frequently diagnosed, accounting for ~68% of breast cancer cases and is very responsive to therapy. In contrast, the HR-/Her2- (triple-negative breast cancer, TNBC) subtype is the most aggressive, which accounts for ~10% of breast cancer cases, and commonly exhibits chemotherapy resistance [1]. Breast cancer commonly develops in the background of chronic diseases such as obesity, diabetes, hypertension, cardiovascular disease, and/or pulmonary disease, decreasing response to therapy and survival [25,26,27,28]. For example, approximately 50–80% of patients with breast cancer have obesity and the prevalence is notably higher in patients with more aggressive cancers [8,29,30]. Obesity is now considered an independent risk factor for the development, morbidity, recurrence, and mortality of breast cancer [6,7,8,9]. Overall, breast cancer is the fifth leading cause of cancer mortality and has the second highest age-standardized mortality rate worldwide, with 685,000 deaths in 2020, accounting for 7% of all cancer deaths and 17% of cancer deaths in women [21].

Mortality is attributable to advanced stage disease and highly metastatic subtypes that poorly respond to currently available targeted drug regimens, displaying high rates of intrinsic or acquired chemotherapy resistance [31]. Breast cancer has a 5-year relative survival rate of 90%, but clinical outcomes vary greatly by stage and subtype [1]. Once the cancer has metastasized and spread to other parts of the body, the 5-year survival rate decreases to 28% [1]. Looking closer at survival by breast cancer subtype, the aggressive TNBC subtype has a 77% 5-year survival rate, but survival decreases to 12% once it has metastasized [1]. In addition to the breast cancer subtype, survival and mortality are greatly impacted by social determinants of health, such as income, food security, and race [32]. Comorbidities worsen cancer survivorship by decreasing quality of life, increasing recurrence and progression, and worsening clinical prognosis [33,34]. For example, women with breast cancer and obesity have an 11% decrease in overall survival [35] and significantly lower treatment efficacy [36]. Surgery, chemotherapy, radiation, endocrine therapies [37], and potentially immunotherapy [38] are less effective in treating breast cancer in people with obesity.

### 2.2. Treatment and Prevention

Cancer is one of the oldest described diseases. Given the location of breast cancer, it has a general ease of detection and is documented in ancient Egyptian medical texts dating to 3000–2500 BCE [39]. First described as an incurable condition, breast cancer has been transformed into a surgically treatable disease and advances in cancer research have resulted in the development of pharmaceutical and radiation treatments, giving rise to an era of targeted therapy. In addition to treatment innovations, screening tools such as improved imaging and genetic testing became available at the end of the 20th Century, highly influencing therapeutic options and efficacy. Treatment regimens for breast cancer may include a combination of surgery, radiation, and pharmacotherapy. Many patients experience multi-organ side-effects of their pharmacotherapies, and this interferes with completion of treatments, decreasing overall potential treatment efficacy. Over 90% of women experience at least one side effect, with nearly half reporting severe or very severe side effects during treatment [40]. Comorbidities, such as obesity and insulin resistance as well as tumor heterogeneity are also major sources of variation in treatment response and resistance [41].

Patients with breast cancer often receive some sort of systemic therapy. Currently, there are over 75 FDA-approved pharmacotherapies for breast cancer [42]. These drugs can be delivered as neoadjuvant and/or adjuvant therapy and a regimen is designed in a specific number of cycles over a set period of time depending on the clinical subtype and stage of disease [43]. For breast cancer, these drugs include chemotherapy, hormonal therapy, tumor-targeted therapy, and immunotherapy, often used in combination [43]. Chemotherapies destroy cancer cells by damaging DNA, interfering with DNA base pairing during replication or transcription, and/or preventing growth and division [44]. However, these processes are not specific to cancer cells, and thus recent advances utilize delivery vehicles for chemotherapy agents to improve cancer cell specificity and lower side effects [45]. Hormonal therapy, also called endocrine therapy, blocks hormone actions and/or lowers hormone levels in the body [44]. Targeted therapies leverage cancer-cell-specific actions based on a genetic mutation, change in protein expression, or the tumor microenvironment (TME) [44]. Immunotherapy, which aims to improve, target, or restore immune system function, has only recently been used for application in breast cancer, compared to other cancer types; most breast tumors are considered immunologically “cold” and viewed to have little potential for effective immunotherapy. In March 2019, the FDA approved the first breast cancer immunotherapy drug, atezolizumab, an immune check-point inhibitor, for treatment of metastatic TNBC [46]. Other types of immunotherapies are currently being investigated with potential for adoptive cell therapy for breast cancer treatment, including TNBC, which can have higher immunogenic peptides than other breast cancer subtypes [47]. The potential combination of nanotechnology and immunotherapy may improve the molecular guidance of the therapy to susceptible breast cancer cells, to overcome tumor microenvironment heterogeneity, and improve the efficacy of the treatment strategy [48].

Despite advances in breast cancer detection, profiling, and treatment, tumors frequently have intrinsic or acquired drug-resistance, decreasing systemic treatment response and increasing breast cancer recurrence [49]. Neoadjuvant chemotherapy (NAT), given before surgical resection, has become increasingly common and results in improved therapeutic response and overall survival with specific improvements in patients with TNBC [50]. Another strategy to improve breast cancer therapeutic response is to use combinations of chemotherapies that have complementary mechanisms of action and can be used synergistically together. An emerging area of breast cancer research is the investigation of therapeutic sensitizers, such as chemosensitizers, to improve cancer cell toxicity to chemotherapy. Chemosensitizers modulate the breast cancer cell’s resistance mechanisms, such as inhibiting the cancer cell’s ability to pump the chemotherapy out of the cell or inactivate the chemotherapy, or by targeting the altered molecules and cell signaling pathways the cancer cell uses to evade death [51]. First-, second-, and third-generation chemosensitizers included calcium channel blockers, immunosuppressive drugs, and microRNAs, but displayed toxicity, low cancer-cell and target specificity, and/or drug-drug interactions in clinical trials [15]. Natural products, including a number of phytochemicals, have gained traction as a source for potential fourth-generation chemosensitizers that can effectively inhibit drug-efflux transporters with limited toxicity [15]. Currently, there are no FDA-approved drugs for use as chemosensitizers, although there are a number of FDA-approved drugs that demonstrate chemosensitizing potential [52], and P-glycoprotein inhibitors remain the lead therapeutic target under clinical investigation [53].

Around 5–10% of all breast cancers are directly hereditary, with ~30% resulting from genetic predisposition, and the remainder from environmental/lifestyle risk factors of cancer and recurrence [22,23,24]. There are multiple risk assessment tools and models, but the Breast Cancer Risk Assessment Tool is the most common model which incorporates age, reproductive status, prior breast cancer disease history, and family breast cancer incidence as factors. Another model is the Tyrer-Cuzick risk model, or IBIS, which is more complex and includes factors such as body mass index (BMI) and extensive personal and family history, but is less accessible and more commonly used in specialized settings [10]. Current FDA-approved chemopreventive medications modulate systemic estrogen production or signaling using aromatase inhibitors or selective estrogen receptor modulators, respectively. These medications are considered for people identified as having a high-risk of developing breast cancer or breast cancer recurrence, but may not be recommended for some women with obesity since blood clots are a known side effect [54]. Other classes of potential chemosensitizers include nuclear and membrane receptors, anti-inflammatory, antioxidant, angiogenesis, and DNA modulation, many of which are investigated through the repurposing of FDA-approved drugs, such as statins, pioglitazone, and metformin [55] or the use of phytochemicals [17].

## 3. Phytochemicals for Breast Cancer Chemoprevention, Therapy, and Sensitization

### 3.1. Phytochemical Anticancer Agents and Application in Breast Cancer Therapy

Natural products, including plants, have known medicinal properties, with use dating to prehistoric times. In contemporary society, natural products serve as bioactive medicines and blueprints for drug development. To this end, more than 60% of cancer drugs approved from 1981 to 2019 stem from natural products [14]. Natural products consist of bioactives from marine and terrestrial microbes and plants. Plants are autotrophic organisms, and thus contain primary and secondary metabolites. Primary metabolites, such as amino acids, nucleotides, sugars, and lipids, are required for plant survival, having direct functions in photosynthesis, respiration, transport, and synthesis of proteins, carbohydrates, or lipids. Conversely, secondary metabolites are compounds produced by the primary metabolic pathways, as well as the subsequent secondary metabolic pathways, and are utilized in the more specialized functions unique to different species of plants. Secondary metabolites classified based on their biosynthetic pathway can be categorized into three groups: (1) nitrogen- and sulfur-containing compounds, (2) phenolic compounds (containing benzene rings with one or more hydroxyl groups), and (3) terpenoids (containing a number of 5-carbon isoprene units). Each of these groups is further divided and subdivided with classification systems based on structure and/or function.

Currently, there are several classes of FDA-approved plant-derived anticancer agents, mostly from the nitrogen-containing alkaloid family, as well as the terpenoid diterpenoid family and the phenolic lignin family (Table 1). Alkaloids can be further classified based on the ring structure moieties they contain, such as isoquinoline or indole compounds, and other modifications they gain through the plant’s diverse metabolic pathways. For example, many indole alkaloids also have isoprene groups, and thus, are referred to by the number of these groups, such as monoterpene indole alkaloids. Other alkaloids are grouped by the Genus of the plant from which they are derived, such as the more well-known vinca alkaloids extracted from the periwinkle plant. Several vinca alkaloids are FDA-approved chemotherapies; specifically, the indole alkaloids vincristine and vinblastine, as well as their derivatives vinorelbine and vindesine, are widely used for treatment in a variety of cancers. Camptothecan analogs, irinotecan and topotecan, are monoterpene indole alkaloids and are also approved chemotherapies that inhibit topoisomerase I [56]. Homoharringtonine, a protein synthesis inhibitor approved to treat tyrosine kinase-resistant chronic myeloid leukemia, is an isoquinoline alkaloid and an ester of cephalotaxane [57]. Epipodophyllotoxins, etoposide, tenisopide, and etopophos, are phenolic lignins that inhibit topoisomerase II [58]. Taxanes, such as paclitaxel and docetaxel, are diterpene terpenoids and some of the most widely use plant-derived chemotherapies [59].

In addition to chemotherapeutic potential, a number of phytochemicals have demonstrated chemosensitizing activities in vitro and in vitro [67]. Chemosensitization is an important clinical strategy to improve the efficacy of chemotherapeutics by decreasing dose-limiting toxicity, improving drug delivery and activation, and overcoming chemoresistance in tumors. Multiple mechanisms for chemoresistance in breast cancer tumors have been identified, which impair drug delivery to and retention within the cancer cell long enough to impart therapeutic effects [49]. In breast cancer, the use of combination chemotherapy regimens aim to circumvent drug resistance, but there are also clinical trials investigating chemosensitization approaches, such as nutritional and exercise interventions [68], as well as novel [69] and repurposed FDA-approved [70] pharmacotherapies. Phytochemicals that display chemosensitization potential include ursolic acid (pentacyclic terpene) [71], bentulinic acid (pentacyclic triterpene) [72], rutin (quercetin glycoside) [73], noscapine (benzylisoquinoline alkaloid) [74], resveratrol (stilbene phenol) [75], curcumin (diarylheptanoid phenol) [76], and genistein (isoflavone) [77] (Table 2).

Phytochemicals are also being studied for their potential as chemopreventive agents. Chemoprevention strategies target multiple points of carcinogenesis and include primary chemoprevention, to block tumor formation, and secondary chemoprevention, to suppress the transition from a benign to a malignant tumor. Primary chemoprevention blocks initiation, the development of a cancer cell through irreversible genetic damage, and promotion, the rise of many daughter cells or clonal expansion of the initial cells containing the mutation [136]. Secondary chemoprevention suppresses promotion, the growth of a tumor, and progression, the irreversible transformation of a benign neoplasm into a malignancy, referred to as malignant conversion [136]. Phytochemicals that display chemopreventive potential include curcumin, resveratrol, tryptanthrin (indoloquinazoline alkaloid), kaempferol (flavonoid), gingerol (aromati ketone), emodin (anthraquinone), quercetin (flavonoid), and genistein (isoflavane) [17] (Table 2). Mechanistic investigations of these phytochemicals demonstrate that they all target multiple points of carcinogenesis, such as initiation, promotion, progression, and inflammation [17].

### 3.2. Mitochondria: Essential Organelles for Breast Cancer Development and Progression

Tumorigenesis, sometimes referred to as carcinogenesis or oncogenesis, is the process of the accumulation of abnormal cells resulting in the formation of a solid tumor or blood-based cancer. Breast cancers are histologically classified as carcinomas, arising from the epithelial component of the breast, or in rare cases (<1%) sarcomas, arising from the stromal connective tissue of the breast [137]. Importantly, the epithelial tissue of the breast consists of different regions that house the beginning, or pre-invasive in situ phases, of cancer that have not yet infiltrated out of the normal breast lobules and ducts into the breast connective tissue. Unfortunately, most breast cancers are not detected in the very early in situ phases and are detected after they have progressed and started to invade the surrounding tissue. Approximately 80% of breast carcinomas are determined to be invasive ductal, and about 10–15% are invasive lobular carcinomas [137]. Cancer development and progression is a dynamic process [138], fluctuating between active and dormant states to enhance cell survival and promote metastasis [139].

Mitochondria are central to cancer cell survival, regulating and supporting key hallmarks of cancer and enabling the cell to respond to evolving needs and changing environments [140]. Approximately 70% of breast tumors display mtDNA mutations and 55% of these mutations are in protein-coding regions, notably in complex I [141]. Consistently, aggressive breast cancers display impairments in expression of multiple oxidative phosphorylation (OXPHOS) complexes [142], specifically in complex I function [143]. Mutations in genes that support mitochondrial structure, functions, and content, have been found to drive carcinogenesis and promote tumor growth as well as support adaptations to changing environments, enabling metabolic flexibility of cancer cells [144,145]. Further, genes that regulate lipid metabolism are upregulated in mammary tissue with minor hyperplasia prior to cancer diagnosis [146] demonstrating the increased mitochondrial fatty acid oxidation observed in breast cancer [147], likely occurring prior to tumor formation. Tumors and their microenvironments are dynamic, consisting of numerous cell types and evolving as the cancer progresses and invades into the surrounding tissue. Cancer severity can be measured by the presence of lymph nodes, vasculature, and immune-cells, as well as epithelial, basal, muscle, and intermediate markers in the cells [148]. Adipocytes, a major component of the TME of mammary tumors, have tumor-promoting effects on breast cancer cells [149,150]. The breast cancer cells, in turn, induce the formation of physically and functionally distinct cancer-associated adipocytes which prime the cancer cells for metastatic potential [149,150,151]. Breast cancer cells utilize adipocyte fatty acids, stimulating mitochondrial fatty acid metabolism, for proliferation and migration [152] as well as invasion [153], critical components of metastatic potential. Metastasis is the spread of cancer cells from the primary site to other locations through the bloodstream or lymph system establishing micro and macro tumors along the way. During the metastatic process, breast cancer cells undergo a range of changes depending on the metastatic site and display unique metabolic signatures with differential shifts in mitochondrial function [154].

Breast cancer metabolic signatures also vary across subtypes [154,155,156], and the major signaling pathways involved in development and progression between the subtypes have been the subject of extensive study [157]. The PI3K/AKT/mTOR pathway is of critical importance for breast cancer survival across subtypes [158]. Specifically, the PI3K/AKT/mTOR pathway has been found to be critical for chemoresistance and survival of TNBC and, although not well studied thus far, is being scrutinized as a potential molecular targeted therapy for TNBC [159]. Metabolic differences between the subtypes and throughout metastatic progression are of interest in examining the response to standard-of-care therapy [160]. Mitochondrial-derived ATP fuels transporter-mediated drug efflux in chemoresistant breast cancer subtypes, whereas glycolytic ATP is dispensable [161]. Mitochondria also play an important role in overcoming chemoresistance through the control of apoptotic signaling. Mitochondrial-related molecules that are targeted for chemosensitization include anti-apoptotic factors such as Bcl-2 [162] and HIF-1α [162]. With new techniques to study cancer metabolism, we may find that many chemosensitizing agents possess either a combination of these changes or other novel metabolic alterations. Additionally, given the role of the TME and tumor progression, regional tissue metabolism and systemic metabolic health are critical components for understanding tumorigenesis.

### 3.3. Phytochemically Targeting Mitochondria

**Figure 1:**
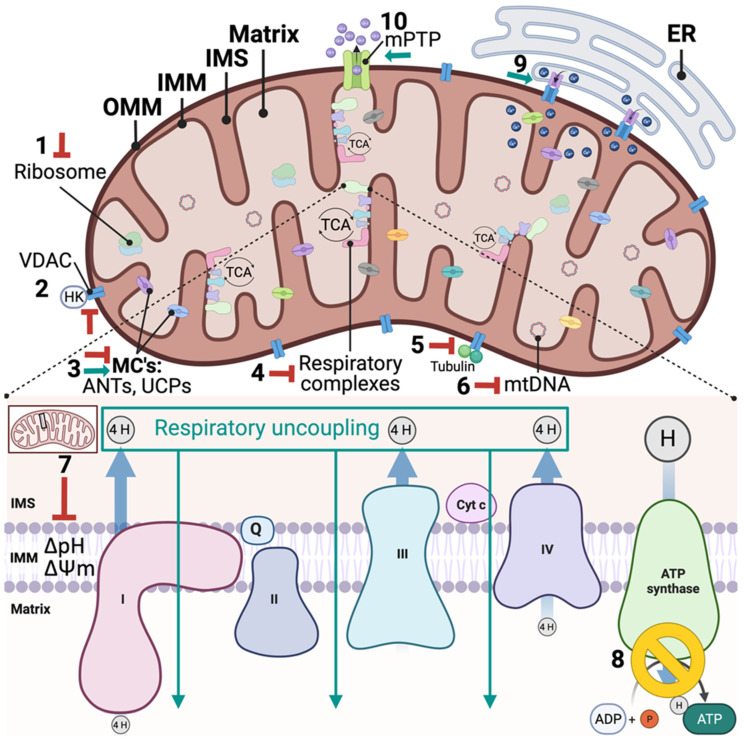
Mitochondrial targets in the anti-cancer effects of phytochemicals. Phytochemicals exert anti-breast cancer effects by targeting various mitochondrial functions, such as: (1) mitochondrial ribosome protein translation, (2) hexokinase-VDAC interaction, (3) metabolite transport (such as inhibition of ANT or upregulation of UCPs), (4) respiratory complex activity, (5) tubulin-VDAC interactions, (6) mtDNA integrity, (7) maintenance of bioenergetic efficiency (ΔpH and ΔΨm) (8) ATP production, (9) ER-calcium influx into the mitochondrial matrix, and (10) intrinsic apoptosis signaling via mPTP-gated cytochrome c release. Abbreviations: OMM, outer mitochondrial membrane; IMM, inner mitochondrial membrane; IMS, inner mitochondrial membrane space; VDAC, voltage-dependent anionic channel; HK, hexokinase; MC’s, metabolite carriers; ANT, Adenine nucleotide translocase; UCP, uncoupling proteins; mtDNA, mitochondrial DNA; ER, endoplasmic reticulum; mPTP, mitochondrial permeability transition pore; ΔpH, pH gradient; ΔΨm, mitochondrial membrane potential gradient.

Mitochondrial-targeted anti-cancer drugs (Mitocans) restrict/inhibit survival via mitochondrial-targeted cellular destabilization. The term was popularized by Neuzil et al. in the early 2000s, classifying drugs that affect major mitochondrial processes, such as hexokinase inhibitors (through interaction with the mitochondrial voltage-dependent anionic channel, VDAC), electron transport/respiratory chain blockers, mPTP activators, and apoptotic inducers (inhibitors of anti-apoptotic proteins and pro-apoptotic mimetics) [163]. The classifications have since been expanded to encompass nine different classes of drugs based on their molecular modes of action. Class 1, hexokinase inhibitors which prevent hexokinase binding to VDAC, disrupt ADP/ATP transport, and promote mitochondrial destabilization; Class 2, Bcl-2-targeting compounds which promote formation of mPTP and apoptosis; Class 3, thiol redox inhibitors, disrupting the anti-oxidant system and promoting mitochondrial damage and apoptosis; Class 4, VDAC and adenine nucleotide translocator (ANT) drugs, which interfere with the transport of critical molecules between the mitochondria and cytosol, such as ADP and ATP; Class 5, compounds targeting the electron transport chain, which can be specific to inhibiting one or more of the five OXPHOS complexes; Class 6, hydrophobic cations targeting the inner mitochondria, which selectively accumulate in the matrix because of the mitochondrial membrane potential (ΔΨm); Class 7, drugs targeting the TCA cycle which inhibit substrate transport or enzyme activity; Class 8, agents that interfere with mtDNA stability and replication, resulting in mtDNA mutation and depletion; and Class 9, drugs targeting other unknown sites within the mitochondria, but are observed to directly impact mitochondrial mechanisms [19,164]. Classes 1–4 target different components on the outer mitochondrial membrane (OMM), Class 5 and Class 6 target the IMM, and Class 8 and Class 9 have mitochondrial matrix targets. Strikingly, most FDA-approved phytochemical anticancer agents, as well as emerging anti-breast cancer phytochemicals, have mitochondrial-targeted mechanisms of action (Figure 1).

Microtubule-targeting agents (MTAs), such as taxanes and Vinca alkaloids, inhibit microtubule dynamics and act as apoptosis inducers. MTAs have been shown to hyperpolarize the mitochondria, resulting in ΔΨm collapse, cytochrome c release, and initiation of the apoptotic cascade [165]. MTAs rapidly impact mitochondrial integrity, affecting stability of the OMM potentially as Class 2 or Class 4 mitocans, but the exact mechanism has not yet been fully elucidated. There are interactions between tubulin and VDACs on the OMM, and this interaction increases VDAC sensitivity and closure [166]; thus, a lack of this interaction via MTAs can induce mitochondrial outer membrane permeability. Additionally, multiple MTAs, such as paclitaxel, have been shown to bind to the apoptotic regulator Bcl-2, inducing mitochondrial outer membrane permeabilization, and intrinsic apoptosis [167]. Supportively, paclitaxel rapidly increases the cell’s cytosolic calcium current by opening the mitochondrial permeability transition pore (mPTP), resulting in a dramatic depletion of mitochondrial calcium prior to robust microtubule disruption [168]. Thus, the mitochondrial mechanism of action of MTAs may be critical for anticancer activity, but further research is required.

Chemotherapy topoisomerase poisons stabilize the topoisomerase-DNA complex in such a way that it can induce breaks, but is not able to ligate the strands, disassociate, and move to another coiled region, resulting in an accumulation of damage. There are two types of isomerases, type I and type II, each with two subtypes expressed in mammals. Like nuclear DNA, mitochondrial DNA maintenance requires the action of four different topoisomerases, with only one exclusive to the mitochondria. The other three are encoded in the nucleus and then function in both nuclear and mitochondrial compartments to maintain DNA topology [169], thus anti-cancer compounds that disrupt mitochondrial topoisomerases act as Class 8 mitocans. Several topoisomerase II poisons, such as the bacterial chemotherapy doxorubicin, have been shown to interact with mitochondrial topoisomerase II, resulting in mtDNA damage and impaired mitochondria [169]. The effect of podophyllotoxins that poison topoisomerase II on mitochondrial topoisomerase II has not been widely studied. The discovery of topoisomerase II purified from mitochondria was validated through the inhibition by both etoposide and teniposide [170], demonstrating that the inhibitors accumulate in the mitochondria and effectively inhibit mitochondrial topoisomerase II. The effective inhibition of mitochondrial topoisomerase by chemotherapy indicates a potential therapeutic mechanism, and etoposide at high doses increases cytochrome c release and decreases calcium ion buffering capacity [171], but has not yet been assessed further to confirm it is able to accumulate in the mitochondria. Conversely, topoisomerase I poisons, such as the camptothecan analog topotecan, are found to accumulate in the mitochondria, inducing mitochondrial DNA-damage and apoptosis [172].

Many chemotherapies effectively target cancer cells through inhibition of ribosomal protein translation. For example, homoharringtonine is a protein translation inhibitor which prevents the initial step of protein synthesis approved for use in patients with chronic myeloid leukemia, which has recently shown preclinical efficacy for the treatment of breast cancer [63]. Although, it did not yield additional benefits to a combination chemotherapy protocol in a clinical trial performed in the 1980s [65]. Ribosomes exist both in the cytosol (80S) as well as in the mitochondria (55S), and it remains unclear if these chemotherapy drugs are targeting mitochondrial and/or cytosolic ribosomes. Homoharringtonin dramatically decreased mitochondrial, but not cytosolic ribosomal protein synthesis at a low dose below the viability IC50 [173]. Homoharringtonin decreased cytosolic ribosomal protein synthesis at a dose 5× higher and was mostly above the viability IC50 for most cell lines [173]. These data possibly indicate that the mitoribosomes are the primary homoharringtonine target for reducing cell viability. Mitoribosomes have a very focused function, to translate 13 proteins that make up the core subunits of the mitochondrial complexes I, III, IV, and V: Complex I- NADH dehydrogenase 1, NADH dehydrogenase 2, NADH dehydrogenase 3, NADH dehydrogenase 4, NADH 4L dehydrogenase, NADH dehydrogenase 5, NADH dehydrogenase 6; Complex III- cytochrome b; Complex IV- cytochrome c oxidase (COX1), cytochrome c oxidase II (COX2), cytochrome c oxidase III (COX3); Complex V- ATP synthase 6 and ATP synthase 8. Thus, anticancer therapies that disrupt mitoribosome protein translation may be considered Class 5 mitocans, because they induce respiratory failure, ATP depletion, and apoptosis.

In addition to FDA-approved anticancer agents, there are other promising anti-breast cancer phytochemicals that destabilize mitochondria as an on-target mechanism of action with chemopreventive and/or chemosensitizing properties. Phytochemicals with promising anticancer mechanisms, like resveratrol, curcumin, quercetin, and capsaicin, are observed to impact mitochondrial respiration and it has been hypothesized that they act as uncouplers [174,175], directly disrupting the proton circuit required for ATP production. Upon further investigation, many of these phytochemicals, such as resveratrol, displayed indirect ways to change the efficiency of coupled ATP production, such as the ability to activate and/or upregulate the expression of uncoupling proteins [92], inhibition of specific complex activity, and disruption of the mitochondrial membrane integrity, and thus, polarization [176]. Conversely, other phytochemicals, such as quercetin [177] and galangin [99], which are flavonoids and hydrophobic weak acids, exhibit direct uncoupling activity. The anticancer activity of Usnic acid and derivatives were first reported in the 1970s and were later demonstrated to be mitochondrial uncouplers [128]. The wide range of mechanisms of action to reduce the available mitochondrial ATP pool, resulting in anti-cancer potential, indicates that it is the mitochondrial ATP the cancers rely on and not the specific way it produces ATP. While many phytochemicals that target the mitochondria effect tumorigenesis and breast cancer progression, few have been clinically tested, such as curcumin, resveratrol, and genistein (Table 2).

Resveratrol is a stilbene phenol that demonstrates efficacy as a chemopreventive agent among women at increased breast cancer risk [87]. In addition to chemoprevention, resveratrol has strong cancer-therapeutic and chemosensitizing effects in breast cancer [89,178] and multiple interrelated mechanisms have been investigated, such as DNA interactions, topoisomerase inhibition, mitochondrial ATPase inhibition, mitochondrial depolarization and respiratory uncoupling, and increased mitochondrial calcium. Resveratrol was recently identified as a novel topoisomerase II inhibitor, preventing ATPase dimerization, and thus, the ATPase activity of the enzyme [179]. Additionally, resveratrol is an inhibitor of mitochondrial complex V, ATP synthase, which blocks the F1 subunit activity [180] and induces calcium overload from the endoplasmic reticulum into the mitochondria [181] due to the increase mitochondrial-ER tethering in breast cancer [182]. An increase in mitochondrial calcium decreases mitochondrial ΔΨm and can lead to mitochondrial permeability transition pore opening, cytochrome c release, and initiation of caspase signaling. Although the direct vs indirect mechanisms are still being elucidated, it is likely that resveratrol localizes to membranes as it is highly lipophilic, indicating a possible increase in trafficking to the mitochondria’s membranes [183] and interaction with mitochondrial ATP synthase, which is a mitochondrial membrane protein.

Curcumin displays multiple levels of anti-cancer effects, demonstrating chemoprotective and chemosensitizing effects, inhibition of breast cancer proliferation, metastases, and angiogenesis and promotion of senescence and apoptosis [83]. Curcumin intravenously delivered was found to be safe, and improved paclitaxel treatment response in metastatic breast cancer patients demonstrating improved efficacy over clinical trials with orally delivered curcumin [79]. Thus far, the mechanisms of action appear to be pleiotropic, which may be highly influenced by where the chemical is able to traffic first within the cancer cell. Curcumin is hydrophobic demonstrating improved stability interacting with lipid membranes [184], has a dissociable proton with a moderate pKa, and displays mitochondrial respiratory uncoupling activity [84]. At lower concentrations, curcumin increases respiration and depolarizes mitochondrial ΔΨm, whereas at higher concentrations it inhibits electron transfer [85]. The localization of curcumin to the mitochondrion is under active investigation and recent findings suggest that it localizes to the endoplasmic reticulum, affecting the mitochondria via ER-mito contacts [185]. Another investigation suggests strong localization of curcumin to the mitochondria within minutes of exposure over a range from 10–100 µM curcumin, resulting in rapid calcium influx into the mitochondrion and sustained ΔΨm [86]. A common challenge with many secondary metabolites is a lack of bioavailability from instability and decreased delivery to targets. Conjugating curcumin to the lipophilic triphenylphosphonium (TPP) cation, facilitated localization to mitochondria and resulted in a 20-fold decrease in IC_50_ to induce cell death in multiple breast cancer cell-lines [82]. A polycurcumin nanoparticle displayed improved efficacy as a chemosensitizer, demonstrating targeted delivery to drug-resistant breast cancer tumors, resulting in dramatic inhibition of tumor growth in combination with chemotherapy [186]. Directly trafficking phytochemicals to cancer cells, as well as directing intracellular localization, offers a promising strategy for targeted therapeutics.

## 4. Challenges and Limitations

Phytochemicals offer an extensive chemical reservoir that is well suited for drug discovery screening and modeling if key challenges can be overcome. A major challenge to studying the anti-cancer potential of phytochemicals is the sourcing of consistently prepared and validated chemicals. Some popular phytochemicals are produced commercially, but others rely on within-lab or program isolation and preparation with a lack of access to phytochemists. Additionally, a limited number of phytochemicals demonstrating promising anti-breast cancer effects have been successfully preclinically tested, and even fewer have made it to clinical trials (Table 2). Most phytochemicals in isolation are not stable, and in vitro do not sufficiently distribute to tissue due to first-pass metabolism and elimination by the gut and the liver. Techniques for improved systemic and tumor delivery can improve bioavailability to enable efficacy testing. The emergence of ‘omics’ and advanced modeling in the phytochemical field will likely improve the efficiency of the drug-discovery pipeline.

In addition to exploring the anti-cancer potential of these phytochemicals, there is a strong public health need to thoroughly study these phytochemicals over the cancer continuum, from primary cancer prevention to treatment of a primary tumor vs metastatic tumors and prevention of recurrence, as well as in the context of standard of care cancer and co-morbidity therapy. Notably, the prevalence of plant medicine use is high in patients with cancer and diabetes [187], and is commonly used in combination with prescribed antineoplastic treatment despite limited information evaluating herb–drug interaction [188]. The likelihood of plant medicine usage is higher in people with a history of obesity [187]. Furthermore, women with breast cancer are most likely to use plant medicines compared to the general population [189,190]. While some medicinal plants are consumed for their purported anti-cancer and immune-boosting potential [191], many consumers are looking for products to reduce chemotherapy side-effects and improve tolerability, and have already been taking the medicines for other health benefits (such as improving metabolic and/or managing mental health), and/or, as cancer survivors, take them to prevent recurrence [192]. These studies indicate a public health need for further study to evaluate potential herb-drug interactions to better inform patient instruction and potentially capitalize on plant-based products as complementary and adjuvant therapies.

## 5. Conclusions

Phytochemicals hold great anti-cancer potential to improve breast cancer survival. FDA-approved phytochemical chemotherapeutic agents demonstrate efficacy in the treatment of breast cancer, with specific promise for the treatment of metastatic breast cancer. These phytochemicals demonstrate mitochondrial targets in their mechanisms of action. Mitochondria also present critical targets both for chemoprevention and chemosensitization. Currently, there are phytochemicals that target mitochondria to prevent cancer, as well as sensitize cancer cells to chemotherapy, under preclinical investigation. Clinical investigation has thus far been limited by compound instability and the low bioavailability that is common with phytochemicals. Advanced data modeling within the phytochemical can help to improve the therapeutic potential of these compounds and/or the synthesis of new compounds using the phytochemicals as bioactive templates.

## Figures and Tables

**Table 1:** FDA-approved phytochemical anti-cancer compounds.

Secondary Metabolite Group	Type	Sub-Class	Phytochemical Example	Use in Breast Cancer
Nitrogen and/or Sulfur containing compounds	Alkaloid	Indole (commonly referred to by Genus: Vinca)	Vincristine, Vinblastin, Vinorelbine, Vindesine	Vinblastine Sulfate-FDA-approved to treat advanced-stage breast cancer Vinorelbine off-label use for metastatic breast cancer [60]
		Monoterpene Indole (Camptothecan analogs)	Irinotecan, Topotecan	*Under pre-clinical and clinical investigation:* Irinotecan displayed therapeutic efficacy in preclinical patient-derived TNBC models [61] Displayed modest clinical efficacy and low tolerability in combination with etoposide in patients with metastatic breast cancer [62]
		Isoquinoline	Homoharringtonine	*Under preclinical investigation:* Inhibited tumor growth in preclinical TNBC models [63] and HR+ model [64] No improved response rate or survival in combination with cyclophosphamide, methotrexate, and fluorouracil in patients with advanced-stage breast cancer [65]
Phenols	Lignan	Aryltetralin (Podophyllotoxins)	Etoposide, Tenisopide, Etopophos	Off-label clinical use for metastatic breast cancer [66] *Under clinical investigation:* Displayed modest clinical efficacy and low tolerability in combination with ironotecan in patients with metastatic breast cancer [62]
Terpenoids	Diterpene	Taxanes	Paclitaxel, Docetaxel	Paclitaxel derivatives and Docetaxel FDA-approved to treat chemoresistant and advanced breast cancer

**Table 2:** Phytochemicals that Display Anti-Breast Cancer Potential Via Mitochondria.

Phytochemical	Examples of Anti-Cancer Evidence	Examples of Mitochondrial Mechanism of Action Evidence
Curcumin	**Chemoprevention** Pre-clinical Efficacy to prevent tumors after carcinogenic exposure [17] In vitro Efficacy to reduce inflammation and tumorigenic signaling in breast epithelial cells [78] **Chemosensitization** Clinical Efficacy with Paclitaxel in MBC [79] Safety with Docetaxel in MBC [80] Pre-clinical Efficacy with a novel [76] and 5-Fluorouracil chemotherapy [81] **Direct anti-cancer action** In vitro Efficacy to induce cell death in multiple breast cancer cell-lines [82] Inhibition of breast cancer proliferation, metastases, and angiogenesis and promotion of senescence and apoptosis [83]	Mitochondrial respiratory uncoupling [84] Increased respiration, decreased ΔΨm, inhibited electron transfer at high concentrations [85] Rapid calcium influx into the mitochondria and sustained decrease in ΔΨm [86]
Resveratrol	**Chemoprevention** Clinical Efficacy to decrease hypermethylation of RASSF-1α in women with increased breast cancer risk [87] In vitro Efficacy as antioxidant [88] **Chemosensitization** In vitro and Pre-clinical Efficacy with talazoparib [75] **Direct anti-cancer action** In vitro and Pre-clinical Efficacy as a phytoestrogen, to decrease proliferation, and to induce apoptosis [89]	Indirect mitochondrial respiratory uncoupling through uncoupling proteins regulation [90]
Kaempferol	**Chemosensitization** In vitro Efficacy with Paclitaxel [91] and Verapamil [92] **Direct anti-cancer action** In vitro and Pre-clinical Efficacy to reduce proliferation [93], induce apoptosis [94], and inhibit metastasis [95]	Modulated BcL-2 protein, decreased ΔΨm, increased caspase signaling [96]
Quercetin	**Chemoprevention** Pre-clinical Efficacy to prevent tumors after carcinogenic exposure [94] **Direct anti-cancer action** In vitro and Pre-clinical Efficacy to inhibit metastasis [97] In vitro Efficacy to inhibit cell proliferation and induce apoptosis [98]	Mitochondrial respiratory uncoupling [99] Increased respiration, decreased ΔΨm, inhibited ANT, opened MPTP, increased cytochrome c release [100] Mitochondrial ATPase F1 inhibitor [101]
Gingerol	**Chemosensitization** Pre-clinical Efficacy with Doxorubicin [102] In vitro Efficacy with Paclitaxel [103] **Chemotolerance** Clinical Efficacy to reduce CINV in BC [104] **Direct anti-cancer action** Pre-clinical Efficacy to induce apoptosis and inhibit metastasis [105] In vitro Efficacy to induce apoptosis [106,107] and induce metastasis [108]	Modulated BcL-2 protein, cytochrome c release, increased caspase signaling [106] Permeabilization of OMM and cytochrome c release [107]
Tryptanthrin	**Chemosensitization** In vitro Efficacy with Doxorubicin [109] **Direct anti-cancer action** Pre-clinical Efficacy to reduce tumor growth and modulate inflammatory TME [110] In vitro Efficacy to induce apoptosis [111]	In a platinum base complex, decreased ΔΨm, initiated cytochrome c-caspase cascade, addition of bromine bond increased localization to the mitochondria [111]
Emodin	**Chemosensitization** Pre-clinical Efficacy with Paclitaxel [112] In vitro Efficacy with Doxorubicin [113], Cisplatin, and Adriamycin [114] **Direct anti-cancer action** Pre-clinical Efficacy to prevent metastasis [115,116] In vitro Efficacy to decrease proliferation [117,118,119]	NADPH- dependent oxidoreductases, decreased complex I proteins, decrease ΔΨm, mitochondrial respiratory uncoupling [117]
Genistein	**Chemoprevention** Clinical Efficacy to reduce proliferation markers in breast tissue of women with high-risk breast cancer risk [120] **Chemosensitization** In vitro Efficacy with Irinotecan metabolite [77], Tamoxifen [121], and Doxorubicin [122] **Direct anti-cancer action** In vitro and Pre-clinical Efficacy to induce cell cycle arrest and apoptosis, regulate cancer-associated microRNAs, decrease proliferation [123]	Modulatedrespiratory complex activity, decreased ΔΨm, cytochrome c-caspase cascade [124]
Ursolic Acid	**Chemosensitization** In vitro Efficacy with rhTRAIL [71] **Direct anti-cancer action** In vitro Efficacy to inhibit metastasis [125]	Activated Cav-1, decreased mitochondrial respiration, decreased ΔΨm, increase apoptosis [125] Hexokinase 2 inhibitor, modulated p53 and mitochondrial ROS [126]
Usnic Acid	**Direct anti-cancer action** In vitro Efficacy to inhibit proliferation [127]	Direct mitochondrial respiratory uncoupler [128]
Bentulinic Acid	**Chemosensitization** In vitro Efficacy with Taxol [72]	Decreased ΔΨm, increased in MPTP [129]
Rutin	**Chemosensitization** In vitro Efficacy with cyclophosphamide [73] **Direct anti-cancer action** In vitro Efficacy to induce apoptosis [130]	Decreased mitochondrial ATP, No effect on isolated mitochondria, needed to be hydrolyzed to quercetin [131]
Noscapine	**Chemosensitization** In vitro and Pre-clinical Efficacy with Docetaxel [74]	Decreased Bax/Bcl-2, induced apoptosis [130]
Capsaicin	**Chemosensitization** In vitro and Pre-clinical Efficacy with paclitaxel [132] **Direct anti-cancer action** In vitro Efficacy to induce apoptosis [133]	Mitochondrial respiratory uncoupling via upregulation of uncoupling proteins [134]
Galangin	**Direct anti-cancer action** In vitro Efficacy to induce apoptosis [135]	Mitochondrial uncoupling and decreased ATP [99]

Abbreviations: MBC, metastatic breast cancer; CINV, chemotherapy-induced nausea and vomiting; ΔΨm, mitochondrial membrane potential; ANT, adenine nucleotide translocase; mPTP, mitochondrial permeability transition pore; OMM, outer mitochondrial membrane; TME, tumor microenvironment; rhTRAIL, recombinant human tumor necrosis factor (TNF)-related apoptosis-inducing ligand.

## Data Availability

Not applicable.

## References

[1] SEER*Explorer: An Interactive Website for SEER Cancer Statistics. https://seer.cancer.gov/explorer/.

[2] Lv L., Yang S., Zhu Y., Zhai X., Li S., Tao X., Dong D. (2022). Relationship between metabolic reprogramming and drug resistance in breast cancer. Front. Oncol..

[3] Germann U.A., Harding M.W. (1995). Chemosensitizers to Overcome and Prevent Multidrug Resistance?. JNCI J. Natl. Cancer Inst..

[4] Woelfel I.A., Fernandez L.J., Idowu M.O., Takabe K. (2018). A high burden of comorbid conditions leads to decreased survival in breast cancer. Gland Surg..

[5] Anwar S.L., Cahyono R., Prabowo D., Avanti W.S., Choridah L., Dwianingsih E.K., Harahap W.A., Aryandono T. (2021). Metabolic comorbidities and the association with risks of recurrent metastatic disease in breast cancer survivors. BMC Cancer.

[6] Reeves G.K., Pirie K., Beral V., Green J., Spencer E., Bull D. (2007). Cancer incidence and mortality in relation to body mass index in the Million Women Study: Cohort study. BMJ.

[7] Renehan A.G., Tyson M., Egger M., Heller R.F., Zwahlen M. (2008). Body-mass index and incidence of cancer: A systematic review and meta-analysis of prospective observational studies. Lancet.

[8] Suzuki R., Orsini N., Saji S., Key T.J., Wolk A. (2009). Body weight and incidence of breast cancer defined by estrogen and progesterone receptor status--a meta-analysis. Int. J. Cancer.

[9] Munsell M.F., Sprague B.L., Berry D.A., Chisholm G., Trentham-Dietz A. (2014). Body mass index and breast cancer risk according to postmenopausal estrogen-progestin use and hormone receptor status. Epidemiol. Rev..

[10] Pruthi S., Heisey R.E., Bevers T.B. (2015). Chemoprevention for Breast Cancer. Ann. Surg. Oncol..

[11] Anderson A.S., Renehan A.G., Saxton J.M., Bell J., Cade J., Cross A.J., King A., Riboli E., Sniehotta F., Treweek S. (2021). Cancer prevention through weight control—Where are we in 2020?. Br. J. Cancer.

[12] Greenwell M., Rahman P.K. (2015). Medicinal Plants: Their Use in Anticancer Treatment. Int. J. Pharm. Sci. Res..

[13] Ntie-Kang F., Telukunta K.K., Fobofou S.A.T., Chukwudi Osamor V., Egieyeh S.A., Valli M., Djoumbou-Feunang Y., Sorokina M., Stork C., Mathai N. (2021). Computational Applications in Secondary Metabolite Discovery (CAiSMD): An online workshop. J. Cheminformatics.

[14] Newman D.J., Cragg G.M. (2020). Natural Products as Sources of New Drugs over the Nearly Four Decades from 01/1981 to 09/2019. J. Nat. Prod..

[15] Hamed A.R., Abdel-Azim N.S., Shams K.A., Hammouda F.M. (2019). Targeting multidrug resistance in cancer by natural chemosensitizers. Bull. Natl. Res. Cent..

[16] Kumar V., Singh D.D., Lakhawat S.S., Yasmeen N., Pandey A., Singla R.K. (2022). Biogenic Phytochemicals Modulating Obesity: From Molecular Mechanism to Preventive and Therapeutic Approaches. Evid. Based Complement. Altern. Med..

[17] Shankar G M., Swetha M., Keerthana C.K., Rayginia T.P., Anto R.J. (2021). Cancer Chemoprevention: A Strategic Approach Using Phytochemicals. Front. Pharm..

[18] Wallace D.C. (2012). Mitochondria and cancer. Nat. Rev. Cancer.

[19] Dong L., Gopalan V., Holland O., Neuzil J. (2020). Mitocans Revisited: Mitochondrial Targeting as Efficient Anti-Cancer Therapy. Int. J. Mol. Sci..

[20] Ferlay J.L.M., Ervik M., Lam F., Colombet M., Mery L., Piñeros M., Znaor A., Soerjomataram I., Bray F. Global Cancer Observatory: Cancer Tomorrow. https://gco.iarc.fr/tomorrow.

[21] Sung H., Ferlay J., Siegel R.L., Laversanne M., Soerjomataram I., Jemal A., Bray F. (2021). Global Cancer Statistics 2020: GLOBOCAN Estimates of Incidence and Mortality Worldwide for 36 Cancers in 185 Countries. CA A Cancer J. Clin..

[22] Bogdanova N., Helbig S., Dörk T. (2013). Hereditary breast cancer: Ever more pieces to the polygenic puzzle. Hered. Cancer Clin. Pract..

[23] Parkin D.M. (2011). The fraction of cancer attributable to lifestyle and environmental factors in the UK in 2010. Br. J. Cancer.

[24] Wu S., Powers S., Zhu W., Hannun Y.A. (2016). Substantial contribution of extrinsic risk factors to cancer development. Nature.

[25] Satariano W.A. (1992). Comorbidity and functional status in older women with breast cancer: Implications for screening, treatment, and prognosis. J. Gerontol..

[26] Knobf M.T. (2007). Psychosocial responses in breast cancer survivors. Semin. Oncol. Nurs..

[27] Fu M.R., Axelrod D., Guth A.A., Cleland C.M., Ryan C.E., Weaver K.R., Qiu J.M., Kleinman R., Scagliola J., Palamar J.J. (2015). Comorbidities and Quality of Life among Breast Cancer Survivors: A Prospective Study. J. Pers. Med..

[28] Ng H.S., Vitry A., Koczwara B., Roder D., McBride M.L. (2019). Patterns of comorbidities in women with breast cancer: A Canadian population-based study. Cancer Causes Control.

[29] Trivers K.F., Lund M.J., Porter P.L., Liff J.M., Flagg E.W., Coates R.J., Eley J.W. (2009). The epidemiology of triple-negative breast cancer, including race. Cancer Causes Control.

[30] Cacho-Díaz B., Spínola-Maroño H., Reynoso N., González-Aguilar A., Mohar-Betancourt A. (2019). Role of Overweight, Obesity, and Comorbidities in the Prognosis of Patients with Breast Cancer with Brain Metastases. Clin. Breast Cancer.

[31] O’Shaughnessy J. (2005). Extending Survival with Chemotherapy in Metastatic Breast Cancer. Oncologist.

[32] Freeman H.P. (2004). Poverty, culture, and social injustice: Determinants of cancer disparities. CA Cancer J. Clin..

[33] Calle E.E., Rodriguez C., Walker-Thurmond K., Thun M.J. (2003). Overweight, obesity, and mortality from cancer in a prospectively studied cohort of U.S. adults. N. Engl. J. Med..

[34] Schmitz K.H., Neuhouser M.L., Agurs-Collins T., Zanetti K.A., Cadmus-Bertram L., Dean L.T., Drake B.F. (2013). Impact of obesity on cancer survivorship and the potential relevance of race and ethnicity. J. Natl. Cancer Inst..

[35] Protani M., Coory M., Martin J.H. (2010). Effect of obesity on survival of women with breast cancer: Systematic review and meta-analysis. Breast Cancer Res. Treat..

[36] Lee K., Kruper L., Dieli-Conwright C.M., Mortimer J.E. (2019). The Impact of Obesity on Breast Cancer Diagnosis and Treatment. Curr. Oncol. Rep..

[37] Darby S., McGale P., Correa C., Taylor C., Arriagada R., Clarke M., Cutter D., Davies C., Ewertz M., Godwin J. (2011). Effect of radiotherapy after breast-conserving surgery on 10-year recurrence and 15-year breast cancer death: Meta-analysis of individual patient data for 10,801 women in 17 randomised trials. Lancet.

[38] Woodall M.J., Neumann S., Campbell K., Pattison S.T., Young S.L. (2020). The Effects of Obesity on Anti-Cancer Immunity and Cancer Immunotherapy. Cancers.

[39] Faguet G.B. (2015). A brief history of cancer: Age-old milestones underlying our current knowledge database. Int. J. Cancer.

[40] Friese C.R., Harrison J.M., Janz N.K., Jagsi R., Morrow M., Li Y., Hamilton A.S., Ward K.C., Kurian A.W., Katz S.J. (2017). Treatment-associated toxicities reported by patients with early-stage invasive breast cancer. Cancer.

[41] Gallo M., Adinolfi V., Barucca V., Prinzi N., Renzelli V., Barrea L., Di Giacinto P., Ruggeri R.M., Sesti F., Arvat E. (2020). Expected and paradoxical effects of obesity on cancer treatment response. Rev. Endocr. Metab. Disord..

[42] FDA Administration Approved Drug Products with Therapeutic Equivalence Evaluations. https://www.accessdata.fda.gov/scripts/cder/ob/index.cfm.

[43] Waks A.G., Winer E.P. (2019). Breast Cancer Treatment: A Review. JAMA.

[44] Korde L.A., Somerfield M.R., Carey L.A., Crews J.R., Denduluri N., Hwang E.S., Khan S.A., Loibl S., Morris E.A., Perez A. (2021). Neoadjuvant Chemotherapy, Endocrine Therapy, and Targeted Therapy for Breast Cancer: ASCO Guideline. J. Clin. Oncol..

[45] Senapati S., Mahanta A.K., Kumar S., Maiti P. (2018). Controlled drug delivery vehicles for cancer treatment and their performance. Signal Transduct. Target. Ther..

[46] Vaddepally R.K., Kharel P., Pandey R., Garje R., Chandra A.B. (2020). Review of Indications of FDA-Approved Immune Checkpoint Inhibitors per NCCN Guidelines with the Level of Evidence. Cancers.

[47] Dees S., Ganesan R., Singh S., Grewal I.S. (2020). Emerging CAR-T Cell Therapy for the Treatment of Triple-Negative Breast Cancer. Mol. Cancer Ther..

[48] Li Y., Miao W., He D., Wang S., Lou J., Jiang Y., Wang S. (2021). Recent Progress on Immunotherapy for Breast Cancer: Tumor Microenvironment, Nanotechnology and More. Front. Bioeng. Biotechnol..

[49] Ji X., Lu Y., Tian H., Meng X., Wei M., Cho W.C. (2019). Chemoresistance mechanisms of breast cancer and their countermeasures. Biomed. Pharmacother..

[50] Spring L.M., Fell G., Arfe A., Sharma C., Greenup R., Reynolds K.L., Smith B.L., Alexander B., Moy B., Isakoff S.J. (2020). Pathologic Complete Response after Neoadjuvant Chemotherapy and Impact on Breast Cancer Recurrence and Survival: A Comprehensive Meta-analysis. Clin. Cancer Res..

[51] Cao J., Zhang M., Wang B., Zhang L., Fang M., Zhou F. (2021). Chemoresistance and Metastasis in Breast Cancer Molecular Mechanisms and Novel Clinical Strategies. Front. Oncol..

[52] Taylor D.J., Parsons C.E., Han H., Jayaraman A., Rege K. (2011). Parallel screening of FDA-approved antineoplastic drugs for identifying sensitizers of TRAIL-induced apoptosis in cancer cells. BMC Cancer.

[53] Lai J.I., Tseng Y.J., Chen M.H., Huang C.F., Chang P.M. (2020). Clinical Perspective of FDA Approved Drugs with P-Glycoprotein Inhibition Activities for Potential Cancer Therapeutics. Front. Oncol..

[54] Force U.P.S.T. (2019). Medication Use to Reduce Risk of Breast Cancer: US Preventive Services Task Force Recommendation Statement. JAMA.

[55] Cazzaniga M., Bonanni B. (2012). Breast cancer chemoprevention: Old and new approaches. J. Biomed. Biotechnol..

[56] Rothenberg M.L. (1997). Topoisomerase I inhibitors: Review and update. Ann. Oncol..

[57] Fresno M., Jiménez A., Vázquez D. (1977). Inhibition of translation in eukaryotic systems by harringtonine. Eur. J. Biochem..

[58] Ross W., Rowe T., Glisson B., Yalowich J., Liu L. (1984). Role of topoisomerase II in mediating epipodophyllotoxin-induced DNA cleavage. Cancer Res..

[59] Cragg G.M., Pezzuto J.M. (2016). Natural Products as a Vital Source for the Discovery of Cancer Chemotherapeutic and Chemopreventive Agents. Med. Princ. Pract..

[60] Hamel S., McNair D.S., Birkett N.J., Mattison D.R., Krantis A., Krewski D. (2015). Off-label use of cancer therapies in women diagnosed with breast cancer in the United States. Springerplus.

[61] Coussy F., El-Botty R., Château-Joubert S., Dahmani A., Montaudon E., Leboucher S., Morisset L., Painsec P., Sourd L., Huguet L. (2020). BRCAness, SLFN11, and RB1 loss predict response to topoisomerase I inhibitors in triple-negative breast cancers. Sci. Transl. Med..

[62] Segar J.M., Reed D., Stopeck A., Livingston R.B., Chalasani P. (2019). A Phase II Study of Irinotecan and Etoposide as Treatment for Refractory Metastatic Breast Cancer. Oncologist.

[63] Yakhni M., Briat A., El Guerrab A., Furtado L., Kwiatkowski F., Miot-Noirault E., Cachin F., Penault-Llorca F., Radosevic-Robin N. (2019). Homoharringtonine, an approved anti-leukemia drug, suppresses triple negative breast cancer growth through a rapid reduction of anti-apoptotic protein abundance. Am. J. Cancer Res..

[64] Wang L.B., Wang D.N., Wu L.G., Cao J., Tian J.H., Liu R., Ma R., Yu J.J., Wang J., Huang Q. (2021). Homoharringtonine inhibited breast cancer cells growth via miR-18a-3p/AKT/mTOR signaling pathway. Int. J. Biol. Sci..

[65] Zhao T.P., Ding G.X., Gao H.Y., Shen Z.Z., Li K.Y. (1986). A clinical trial of homoharringtonine in the treatment of advanced breast cancer. Tumori.

[66] Giannone G., Milani A., Ghisoni E., Genta S., Mittica G., Montemurro F., Valabrega G. (2018). Oral etoposide in heavily pre-treated metastatic breast cancer: A retrospective series. Breast.

[67] Prasad N.R., Muthusamy G., Shanmugam M., Ambudkar S.V. (2016). South Asian Medicinal Compounds as Modulators of Resistance to Chemotherapy and Radiotherapy. Cancers.

[68] Kirkham A.A., King K., Joy A.A., Pelletier A.B., Mackey J.R., Young K., Zhu X., Meza-Junco J., Basi S.K., Hiller J.P. (2021). Rationale and design of the Diet Restriction and Exercise-induced Adaptations in Metastatic breast cancer (DREAM) study: A 2-arm, parallel-group, phase II, randomized control trial of a short-term, calorie-restricted, and ketogenic diet plus exercise during intravenous chemotherapy versus usual care. BMC Cancer.

[69] Muttiah C., Whittle J.R., Oakman C., Lindeman G.J. (2022). PALVEN: Phase Ib trial of palbociclib, letrozole and venetoclax in estrogen receptor- and BCL2-positive advanced breast cancer. Future Oncol..

[70] Zhang Z., Zhou L., Xie N., Nice E.C., Zhang T., Cui Y., Huang C. (2020). Overcoming cancer therapeutic bottleneck by drug repurposing. Signal Transduct. Target. Ther..

[71] Manouchehri J.M., Kalafatis M. (2018). Ursolic Acid Promotes the Sensitization of rhTRAIL-resistant Triple-negative Breast Cancer. Anticancer Res..

[72] Cai Y., Zheng Y., Gu J., Wang S., Wang N., Yang B., Zhang F., Wang D., Fu W., Wang Z. (2018). Betulinic acid chemosensitizes breast cancer by triggering ER stress-mediated apoptosis by directly targeting GRP78. Cell Death Dis..

[73] Iriti M., Kubina R., Cochis A., Sorrentino R., Varoni E.M., Kabała-Dzik A., Azzimonti B., Dziedzic A., Rimondini L., Wojtyczka R.D. (2017). Rutin, a Quercetin Glycoside, Restores Chemosensitivity in Human Breast Cancer Cells. Phytother Res..

[74] Doddapaneni R., Patel K., Chowdhury N., Singh M. (2017). Reversal of drug-resistance by noscapine chemo-sensitization in docetaxel resistant triple negative breast cancer. Sci. Rep..

[75] Pai Bellare G., Sankar Patro B. (2022). Resveratrol sensitizes breast cancer to PARP inhibitor, talazoparib through dual inhibition of AKT and autophagy flux. Biochem. Pharmacol..

[76] Shao C., Wu J., Han S., Liu Y., Su Z., Zhu H.-L., Liu H.-K., Qian Y. (2022). Biotinylated curcumin as a novel chemosensitizer enhances naphthalimide-induced autophagic cell death in breast cancer cells. Eur. J. Med. Chem..

[77] Lee H.-J., Choi C.-H. (2022). Characterization of SN38-resistant T47D breast cancer cell sublines overexpressing BCRP, MRP1, MRP2, MRP3, and MRP4. BMC Cancer.

[78] Vaughan R.A., Garcia-Smith R., Dorsey J., Griffith J.K., Bisoffi M., Trujillo K.A. (2013). Tumor necrosis factor alpha induces Warburg-like metabolism and is reversed by anti-inflammatory curcumin in breast epithelial cells. Int. J. Cancer.

[79] Saghatelyan T., Tananyan A., Janoyan N., Tadevosyan A., Petrosyan H., Hovhannisyan A., Hayrapetyan L., Arustamyan M., Arnhold J., Rotmann A.R. (2020). Efficacy and safety of curcumin in combination with paclitaxel in patients with advanced, metastatic breast cancer: A comparative, randomized, double-blind, placebo-controlled clinical trial. Phytomedicine.

[80] Bayet-Robert M., Kwiatkowski F., Leheurteur M., Gachon F., Planchat E., Abrial C., Mouret-Reynier M.A., Durando X., Barthomeuf C., Chollet P. (2010). Phase I dose escalation trial of docetaxel plus curcumin in patients with advanced and metastatic breast cancer. Cancer Biol. Ther..

[81] Haritha N.H., Nawab A., Vijayakurup V., Anto N.P., Liju V.B., Alex V.V., Amrutha A.N., Aiswarya S.U., Swetha M., Vinod B.S. (2021). Targeting Thymidylate Synthase Enhances the Chemosensitivity of Triple-Negative Breast Cancer Towards 5-FU-Based Combinatorial Therapy. Front. Oncol..

[82] Reddy C.A., Somepalli V., Golakoti T., Kanugula A.K., Karnewar S., Rajendiran K., Vasagiri N., Prabhakar S., Kuppusamy P., Kotamraju S. (2014). Mitochondrial-Targeted Curcuminoids: A Strategy to Enhance Bioavailability and Anticancer Efficacy of Curcumin. PLoS ONE.

[83] Banik U., Parasuraman S., Adhikary A.K., Othman N.H. (2017). Curcumin: The spicy modulator of breast carcinogenesis. J. Exp. Clin. Cancer Res..

[84] Lim H.W., Lim H.Y., Wong K.P. (2009). Uncoupling of oxidative phosphorylation by curcumin: Implication of its cellular mechanism of action. Biochem. Biophys. Res. Commun..

[85] Moustapha A., Pérétout P.A., Rainey N.E., Sureau F., Geze M., Petit J.M., Dewailly E., Slomianny C., Petit P.X. (2015). Curcumin induces crosstalk between autophagy and apoptosis mediated by calcium release from the endoplasmic reticulum, lysosomal destabilization and mitochondrial events. Cell Death Discov..

[86] Olivas-Aguirre M., Torres-López L., Pottosin I., Dobrovinskaya O. (2021). Phenolic Compounds Cannabidiol, Curcumin and Quercetin Cause Mitochondrial Dysfunction and Suppress Acute Lymphoblastic Leukemia Cells. Int. J. Mol. Sci..

[87] Zhu W., Qin W., Zhang K., Rottinghaus G.E., Chen Y.-C., Kliethermes B., Sauter E.R. (2012). Trans-Resveratrol Alters Mammary Promoter Hypermethylation in Women at Increased Risk for Breast Cancer. Nutr. Cancer.

[88] Chatterjee A., Ronghe A., Padhye S.B., Spade D.A., Bhat N.K., Bhat H.K. (2018). Antioxidant activities of novel resveratrol analogs in breast cancer. J. Biochem. Mol. Toxicol..

[89] Sinha D., Sarkar N., Biswas J., Bishayee A. (2016). Resveratrol for breast cancer prevention and therapy: Preclinical evidence and molecular mechanisms. Semin. Cancer Biol..

[90] Gibellini L., Bianchini E., De Biasi S., Nasi M., Cossarizza A., Pinti M. (2015). Natural Compounds Modulating Mitochondrial Functions. Evid.-Based Complement. Altern. Med..

[91] Aghazadeh T., Bakhtiari N., Rad I.A., Ramezani F. (2021). Formulation of Kaempferol in Nanostructured Lipid Carriers (NLCs): A Delivery Platform to Sensitization of MDA-MB468 Breast Cancer Cells to Paclitaxel. Biointerface Res. Appl. Chem..

[92] Nandi S.K., Roychowdhury T., Chattopadhyay S., Basu S., Chatterjee K., Choudhury P., Banerjee N., Saha P., Mukhopadhyay S., Mukhopadhyay A. (2022). Deregulation of the CD44-NANOG-MDR1 associated chemoresistance pathways of breast cancer stem cells potentiates the anti-cancer effect of Kaempferol in synergism with Verapamil. Toxicol. Appl. Pharm..

[93] Kim S.H., Hwang K.A., Choi K.C. (2016). Treatment with kaempferol suppresses breast cancer cell growth caused by estrogen and triclosan in cellular and xenograft breast cancer models. J. Nutr. Biochem..

[94] Verma A.K., Johnson J.A., Gould M.N., Tanner M.A. (1988). Inhibition of 7,12-dimethylbenz(a)anthracene- and N-nitrosomethylurea-induced rat mammary cancer by dietary flavonol quercetin. Cancer Res..

[95] Chen A.Y., Chen Y.C. (2013). A review of the dietary flavonoid, kaempferol on human health and cancer chemoprevention. Food Chem..

[96] Zhu L., Xue L. (2019). Kaempferol Suppresses Proliferation and Induces Cell Cycle Arrest, Apoptosis, and DNA Damage in Breast Cancer Cells. Oncol. Res..

[97] Li X., Zhou N., Wang J., Liu Z., Wang X., Zhang Q., Liu Q., Gao L., Wang R. (2018). Quercetin suppresses breast cancer stem cells (CD44(+)/CD24(-)) by inhibiting the PI3K/Akt/mTOR-signaling pathway. Life Sci..

[98] Seo H.S., Ku J.M., Choi H.S., Choi Y.K., Woo J.K., Kim M., Kim I., Na C.H., Hur H., Jang B.H. (2016). Quercetin induces caspase-dependent extrinsic apoptosis through inhibition of signal transducer and activator of transcription 3 signaling in HER2-overexpressing BT-474 breast cancer cells. Oncol. Rep..

[99] Dorta D.J., Pigoso A.A., Mingatto F.E., Rodrigues T., Prado I.M.R., Helena A.F.C., Uyemura S.A., Santos A.C., Curti C. (2005). The interaction of flavonoids with mitochondria: Effects on energetic processes. Chem.-Biol. Interact..

[100] Ortega R., García N. (2009). The flavonoid quercetin induces changes in mitochondrial permeability by inhibiting adenine nucleotide translocase. J. Bioenerg. Biomembr..

[101] Lang D.R., Racker E. (1974). Effects of quercetin and F1 inhibitor on mitochondrial ATPase and energy-linked reactions in submitochondrial particles. Biochim. Et Biophys. Acta (BBA)-Bioenerg..

[102] Baptista Moreno Martin A.C., Tomasin R., Luna-Dulcey L., Graminha A.E., Araújo Naves M., Teles R.H.G., da Silva V.D., da Silva J.A., Vieira P.C., Annabi B. (2020). [10]-Gingerol improves doxorubicin anticancer activity and decreases its side effects in triple negative breast cancer models. Cell Oncol. (Dordr).

[103] Wala K., Szlasa W., Sauer N., Kasperkiewicz-Wasilewska P., Szewczyk A., Saczko J., Rembiałkowska N., Kulbacka J., Baczyńska D. (2022). Anticancer Efficacy of 6-Gingerol with Paclitaxel against Wild Type of Human Breast Adenocarcinoma. Molecules.

[104] Konmun J., Danwilai K., Ngamphaiboon N., Sripanidkulchai B., Sookprasert A., Subongkot S. (2017). A phase II randomized double-blind placebo-controlled study of 6-gingerol as an anti-emetic in solid tumor patients receiving moderately to highly emetogenic chemotherapy. Med. Oncol..

[105] Martin A., Fuzer A.M., Becceneri A.B., da Silva J.A., Tomasin R., Denoyer D., Kim S.H., McIntyre K.A., Pearson H.B., Yeo B. (2017). [10]-Gingerol induces apoptosis and inhibits metastatic dissemination of triple negative breast cancer in vivo. Oncotarget.

[106] Sp N., Kang D.Y., Lee J.M., Bae S.W., Jang K.J. (2021). Potential Antitumor Effects of 6-Gingerol in p53-Dependent Mitochondrial Apoptosis and Inhibition of Tumor Sphere Formation in Breast Cancer Cells. Int. J. Mol. Sci..

[107] Bernard M.M., McConnery J.R., Hoskin D.W. (2017). [10]-Gingerol, a major phenolic constituent of ginger root, induces cell cycle arrest and apoptosis in triple-negative breast cancer cells. Exp. Mol. Pathol..

[108] Lee H.S., Seo E.Y., Kang N.E., Kim W.K. (2008). [6]-Gingerol inhibits metastasis of MDA-MB-231 human breast cancer cells. J. Nutr. Biochem..

[109] Yu S.T., Chen T.M., Tseng S.Y., Chen Y.H. (2007). Tryptanthrin inhibits MDR1 and reverses doxorubicin resistance in breast cancer cells. Biochem. Biophys. Res. Commun..

[110] Zeng Q., Luo C., Cho J., Lai D., Shen X., Zhang X., Zhou W. (2021). Tryptanthrin exerts anti-breast cancer effects both in vitro and in vivo through modulating the inflammatory tumor microenvironment. Acta Pharm..

[111] Qin Q.P., Zou B.Q., Hu F.L., Huang G.B., Wang S.L., Gu Y.Q., Tan M.X. (2018). Platinum(ii) complexes with rutaecarpine and tryptanthrin derivatives induce apoptosis by inhibiting telomerase activity and disrupting mitochondrial function. Medchemcomm.

[112] Zhang L., Lau Y.K., Xia W., Hortobagyi G.N., Hung M.C. (1999). Tyrosine kinase inhibitor emodin suppresses growth of HER-2/neu-overexpressing breast cancer cells in athymic mice and sensitizes these cells to the inhibitory effect of paclitaxel. Clin. Cancer Res..

[113] Li B., Zhao X., Zhang L., Cheng W. (2020). Emodin Interferes with AKT1-Mediated DNA Damage and Decreases Resistance of Breast Cancer Cells to Doxorubicin. Front. Oncol..

[114] Fu J.M., Zhou J., Shi J., Xie J.S., Huang L., Yip A.Y., Loo W.T., Chow L.W., Ng E.L. (2012). Emodin affects ERCC1 expression in breast cancer cells. J. Transl. Med..

[115] Jia X., Yu F., Wang J., Iwanowycz S., Saaoud F., Wang Y., Hu J., Wang Q., Fan D. (2014). Emodin suppresses pulmonary metastasis of breast cancer accompanied with decreased macrophage recruitment and M2 polarization in the lungs. Breast Cancer Res. Treat..

[116] Liu Q., Hodge J., Wang J., Wang Y., Wang L., Singh U., Li Y., Yao Y., Wang D., Ai W. (2020). Emodin reduces Breast Cancer Lung Metastasis by suppressing Macrophage-induced Breast Cancer Cell Epithelial-mesenchymal transition and Cancer Stem Cell formation. Theranostics.

[117] Dumit V.I., Zerbes R.M., Kaeser-Pebernard S., Rackiewicz M., Wall M.T., Gretzmeier C., Küttner V., van der Laan M., Braun R.J., Dengjel J. (2017). Respiratory status determines the effect of emodin on cell viability. Oncotarget.

[118] Zhang L., Chang C.J., Bacus S.S., Hung M.C. (1995). Suppressed transformation and induced differentiation of HER-2/neu-overexpressing breast cancer cells by emodin. Cancer Res..

[119] Akkol E.K., Tatlı I.I., Karatoprak G., Ağar O.T., Yücel Ç., Sobarzo-Sánchez E., Capasso R. (2021). Is Emodin with Anticancer Effects Completely Innocent? Two Sides of the Coin. Cancers.

[120] Khan S.A., Chatterton R.T., Michel N., Bryk M., Lee O., Ivancic D., Heinz R., Zalles C.M., Helenowski I.B., Jovanovic B.D. (2012). Soy isoflavone supplementation for breast cancer risk reduction: A randomized phase II trial. Cancer Prev. Res. (Phila).

[121] Mai Z., Blackburn G.L., Zhou J.R. (2007). Genistein sensitizes inhibitory effect of tamoxifen on the growth of estrogen receptor-positive and HER2-overexpressing human breast cancer cells. Mol. Carcinog.

[122] Xue J.P., Wang G., Zhao Z.B., Wang Q., Shi Y. (2014). Synergistic cytotoxic effect of genistein and doxorubicin on drug-resistant human breast cancer MCF-7/Adr cells. Oncol. Rep..

[123] Bhat S.S., Prasad S.K., Shivamallu C., Prasad K.S., Syed A., Reddy P., Cull C.A., Amachawadi R.G. (2021). Genistein: A Potent Anti-Breast Cancer Agent. Curr. Issues Mol. Biol..

[124] de Oliveira M.R. (2016). Evidence for genistein as a mitochondriotropic molecule. Mitochondrion.

[125] Wang S., Chang X., Zhang J., Li J., Wang N., Yang B., Pan B., Zheng Y., Wang X., Ou H. (2021). Ursolic Acid Inhibits Breast Cancer Metastasis by Suppressing Glycolytic Metabolism via Activating SP1/Caveolin-1 Signaling. Front. Oncol..

[126] Feng X.M., Su X.L. (2019). Anticancer effect of ursolic acid via mitochondria-dependent pathways. Oncol. Lett.

[127] Bazin M.-A., Lamer A.-C.L., Delcros J.-G., Rouaud I., Uriac P., Boustie J., Corbel J.-C., Tomasi S. (2008). Synthesis and cytotoxic activities of usnic acid derivatives. Bioorganic Med. Chem..

[128] Abo-Khatwa A.N., al-Robai A.A., al-Jawhari D.A. (1996). Lichen acids as uncouplers of oxidative phosphorylation of mouse-liver mitochondria. Nat. Toxins.

[129] Fulda S., Scaffidi C., Susin S.A., Krammer P.H., Kroemer G., Peter M.E., Debatin K.M. (1998). Activation of mitochondria and release of mitochondrial apoptogenic factors by betulinic acid. J. Biol. Chem..

[130] Quisbert-Valenzuela E.O., Calaf G.M. (2016). Apoptotic effect of noscapine in breast cancer cell lines. Int. J. Oncol..

[131] Takahashi L., Sert M.A., Kelmer-Bracht A.M., Bracht A., Ishii-Iwamoto E.L. (1998). Effects of rutin and quercetin on mitochondrial metabolism and on ATP levels in germinating tissues of Glycine max. Plant Physiol. Biochem..

[132] Lan Y., Sun Y., Yang T., Ma X., Cao M., Liu L., Yu S., Cao A., Liu Y. (2019). Co-Delivery of Paclitaxel by a Capsaicin Prodrug Micelle Facilitating for Combination Therapy on Breast Cancer. Mol. Pharm..

[133] Thoennissen N.H., O’Kelly J., Lu D., Iwanski G.B., La D.T., Abbassi S., Leiter A., Karlan B., Mehta R., Koeffler H.P. (2010). Capsaicin causes cell-cycle arrest and apoptosis in ER-positive and -negative breast cancer cells by modulating the EGFR/HER-2 pathway. Oncogene.

[134] Masuda Y., Haramizu S., Oki K., Ohnuki K., Watanabe T., Yazawa S., Kawada T., Hashizume S.-i., Fushiki T. (2003). Upregulation of uncoupling proteins by oral administration of capsiate, a nonpungent capsaicin analog. J. Appl. Physiol..

[135] Song W., Yan C.-Y., Zhou Q.-Q., Zhen L.-L. (2017). Galangin potentiates human breast cancer to apoptosis induced by TRAIL through activating AMPK. Biomed. Pharmacother..

[136] Weston Ainsley H.C.C., Kufe D.W.P.R., Weichselbaum R.R. (2003). Multistage Carcinogenesis. Holland-Frei Cancer Medicine.

[137] Feng Y., Spezia M., Huang S., Yuan C., Zeng Z., Zhang L., Ji X., Liu W., Huang B., Luo W. (2018). Breast cancer development and progression: Risk factors, cancer stem cells, signaling pathways, genomics, and molecular pathogenesis. Genes Dis..

[138] Hlatky L., Hahnfeldt P. (2014). Beyond the cancer cell: Progression-level determinants highlight the multiscale nature of carcinogenesis risk. Cancer Res..

[139] Park S.-Y., Nam J.-S. (2020). The force awakens: Metastatic dormant cancer cells. Exp. Mol. Med..

[140] Giampazolias E., Tait S.W. (2016). Mitochondria and the hallmarks of cancer. FEBS J..

[141] McMahon S., LaFramboise T. (2014). Mutational patterns in the breast cancer mitochondrial genome, with clinical correlates. Carcinogenesis.

[142] Putignani L., Raffa S., Pescosolido R., Rizza T., Del Chierico F., Leone L., Aimati L., Signore F., Carrozzo R., Callea F. (2012). Preliminary evidences on mitochondrial injury and impaired oxidative metabolism in breast cancer. Mitochondrion.

[143] Zunica E.R.M., Axelrod C.L., Cho E., Spielmann G., Davuluri G., Alexopoulos S.J., Beretta M., Hoehn K.L., Dantas W.S., Stadler K. (2021). Breast cancer growth and proliferation is suppressed by the mitochondrial targeted furazano [3,4-b]pyrazine BAM15. Cancer Metab..

[144] Brandon M., Baldi P., Wallace D.C. (2006). Mitochondrial mutations in cancer. Oncogene.

[145] Moindjie H., Rodrigues-Ferreira S., Nahmias C. (2021). Mitochondrial Metabolism in Carcinogenesis and Cancer Therapy. Cancers.

[146] Marino N., German R., Rao X., Simpson E., Liu S., Wan J., Liu Y., Sandusky G., Jacobsen M., Stoval M. (2020). Upregulation of lipid metabolism genes in the breast prior to cancer diagnosis. NPJ Breast Cancer.

[147] Li Y.-J., Fahrmann J.F., Aftabizadeh M., Zhao Q., Tripathi S.C., Zhang C., Yuan Y., Ann D., Hanash S., Yu H. (2022). Fatty acid oxidation protects cancer cells from apoptosis by increasing mitochondrial membrane lipids. Cell Rep..

[148] Liu H. (2014). Application of Immunohistochemistry in Breast Pathology: A Review and Update. Arch. Pathol. Lab. Med..

[149] Dirat B., Bochet L., Dabek M., Daviaud D., Dauvillier S., Majed B., Wang Y.Y., Meulle A., Salles B., Le Gonidec S. (2011). Cancer-associated adipocytes exhibit an activated phenotype and contribute to breast cancer invasion. Cancer Res..

[150] Iyengar P., Espina V., Williams T.W., Lin Y., Berry D., Jelicks L.A., Lee H., Temple K., Graves R., Pollard J. (2005). Adipocyte-derived collagen VI affects early mammary tumor progression in vivo, demonstrating a critical interaction in the tumor/stroma microenvironment. J. Clin. Investig..

[151] Andarawewa K.L., Motrescu E.R., Chenard M.P., Gansmuller A., Stoll I., Tomasetto C., Rio M.C. (2005). Stromelysin-3 is a potent negative regulator of adipogenesis participating to cancer cell-adipocyte interaction/crosstalk at the tumor invasive front. Cancer Res..

[152] Balaban S., Shearer R.F., Lee L.S., van Geldermalsen M., Schreuder M., Shtein H.C., Cairns R., Thomas K.C., Fazakerley D.J., Grewal T. (2017). Adipocyte lipolysis links obesity to breast cancer growth: Adipocyte-derived fatty acids drive breast cancer cell proliferation and migration. Cancer Metab..

[153] Wang Y.Y., Attané C., Milhas D., Dirat B., Dauvillier S., Guerard A., Gilhodes J., Lazar I., Alet N., Laurent V. (2017). Mammary adipocytes stimulate breast cancer invasion through metabolic remodeling of tumor cells. JCI Insight.

[154] Dupuy F., Tabariès S., Andrzejewski S., Dong Z., Blagih J., Annis M.G., Omeroglu A., Gao D., Leung S., Amir E. (2015). PDK1-Dependent Metabolic Reprogramming Dictates Metastatic Potential in Breast Cancer. Cell Metab..

[155] Sarmiento-Salinas F.L., Delgado-Magallón A., Montes-Alvarado J.B., Ramírez-Ramírez D., Flores-Alonso J.C., Cortés-Hernández P., Reyes-Leyva J., Herrera-Camacho I., Anaya-Ruiz M., Pelayo R. (2019). Breast Cancer Subtypes Present a Differential Production of Reactive Oxygen Species (ROS) and Susceptibility to Antioxidant Treatment. Front. Oncol..

[156] Haukaas T.H., Euceda L.R., Giskeødegård G.F., Lamichhane S., Krohn M., Jernström S., Aure M.R., Lingjærde O.C., Schlichting E., Garred Ø. (2016). Metabolic clusters of breast cancer in relation to gene- and protein expression subtypes. Cancer Metab..

[157] Dai X., Xiang L., Li T., Bai Z. (2016). Cancer Hallmarks, Biomarkers and Breast Cancer Molecular Subtypes. J. Cancer.

[158] Miricescu D., Totan A., Stanescu-Spinu I.-I., Badoiu S.C., Stefani C., Greabu M. (2020). PI3K/AKT/mTOR Signaling Pathway in Breast Cancer: From Molecular Landscape to Clinical Aspects. Int. J. Mol. Sci..

[159] Khan M.A., Jain V.K., Rizwanullah M., Ahmad J., Jain K. (2019). PI3K/AKT/mTOR pathway inhibitors in triple-negative breast cancer: A review on drug discovery and future challenges. Drug Discov. Today.

[160] Gandhi N., Das G.M. (2019). Metabolic Reprogramming in Breast Cancer and Its Therapeutic Implications. Cells.

[161] Giddings E.L., Champagne D.P., Wu M.-H., Laffin J.M., Thornton T.M., Valenca-Pereira F., Culp-Hill R., Fortner K.A., Romero N., East J. (2021). Mitochondrial ATP fuels ABC transporter-mediated drug efflux in cancer chemoresistance. Nat. Commun..

[162] Tekedereli I., Alpay S.N., Akar U., Yuca E., Ayugo-Rodriguez C., Han H.-D., Sood A.K., Lopez-Berestein G., Ozpolat B. (2013). Therapeutic Silencing of Bcl-2 by Systemically Administered siRNA Nanotherapeutics Inhibits Tumor Growth by Autophagy and Apoptosis and Enhances the Efficacy of Chemotherapy in Orthotopic Xenograft Models of ER (−) and ER (+) Breast Cancer. Mol. Ther.-Nucleic Acids.

[163] Ralph S.J., Low P., Dong L., Lawen A., Neuzil J. (2006). Mitocans: Mitochondrial targeted anti-cancer drugs as improved therapies and related patent documents. Recent Pat. Anticancer Drug Discov..

[164] Neuzil J., Dong L.F., Rohlena J., Truksa J., Ralph S.J. (2013). Classification of mitocans, anti-cancer drugs acting on mitochondria. Mitochondrion.

[165] Rovini A., Savry A., Braguer D., Carré M. (2011). Microtubule-targeted agents: When mitochondria become essential to chemotherapy. Biochim. Et Biophys. Acta (BBA)-Bioenerg..

[166] Rostovtseva T.K., Sheldon K.L., Hassanzadeh E., Monge C., Saks V., Bezrukov S.M., Sackett D.L. (2008). Tubulin binding blocks mitochondrial voltage-dependent anion channel and regulates respiration. Proc. Natl. Acad. Sci. USA.

[167] Ferlini C., Cicchillitti L., Raspaglio G., Bartollino S., Cimitan S., Bertucci C., Mozzetti S., Gallo D., Persico M., Fattorusso C. (2009). Paclitaxel Directly Binds to Bcl-2 and Functionally Mimics Activity of Nur77. Cancer Res..

[168] Kidd J.F., Pilkington M.F., Schell M.J., Fogarty K.E., Skepper J.N., Taylor C.W., Thorn P. (2002). Paclitaxel Affects Cytosolic Calcium Signals by Opening the Mitochondrial Permeability Transition Pore *. J. Biol. Chem..

[169] Goffart S., Hangas A., Pohjoismäki J.L.O. (2019). Twist and Turn-Topoisomerase Functions in Mitochondrial DNA Maintenance. Int. J. Mol. Sci..

[170] Lin J.H., Castora F.J. (1991). DNA topoisomerase II from mammalian mitochondria is inhibited by the antitumor drugs, m-AMSA and VM-26. Biochem. Biophys. Res. Commun..

[171] Robertson J.D., Gogvadze V., Zhivotovsky B., Orrenius S. (2000). Distinct Pathways for Stimulation of Cytochrome cRelease by Etoposide*. J. Biol. Chem..

[172] de la Loza M.C.D., Wellinger R.E. (2009). A novel approach for organelle-specific DNA damage targeting reveals different susceptibility of mitochondrial DNA to the anticancer drugs camptothecin and topotecan. Nucleic Acids Res..

[173] Myasnikov A.G., Kundhavai Natchiar S., Nebout M., Hazemann I., Imbert V., Khatter H., Peyron J.F., Klaholz B.P. (2016). Structure-function insights reveal the human ribosome as a cancer target for antibiotics. Nat. Commun..

[174] Grabacka M.M., Gawin M., Pierzchalska M. (2014). Phytochemical modulators of mitochondria: The search for chemopreventive agents and supportive therapeutics. Pharmaceuticals.

[175] Pratheeshkumar P., Sreekala C., Zhang Z., Budhraja A., Ding S., Son Y.-O., Wang X., Hitron A., Hyun-Jung K., Wang L. (2012). Cancer prevention with promising natural products: Mechanisms of action and molecular targets. Anticancer Agents Med. Chem..

[176] Stevens J.F., Revel J.S., Maier C.S. (2018). Mitochondria-Centric Review of Polyphenol Bioactivity in Cancer Models. Antioxid. Redox Signal.

[177] van Dijk C., Driessen A.J., Recourt K. (2000). The uncoupling efficiency and affinity of flavonoids for vesicles. Biochem. Pharm..

[178] Cocetta V., Quagliariello V., Fiorica F., Berretta M., Montopoli M. (2021). Resveratrol as Chemosensitizer Agent: State of Art and Future Perspectives. Int. J. Mol. Sci..

[179] Lee J.H., Wendorff T.J., Berger J.M. (2017). Resveratrol: A novel type of topoisomerase II inhibitor. J. Biol. Chem..

[180] Gledhill J.R., Walker J.E. (2005). Inhibition sites in F1-ATPase from bovine heart mitochondria. Biochem. J..

[181] Madreiter-Sokolowski C.T., Gottschalk B., Parichatikanond W., Eroglu E., Klec C., Waldeck-Weiermair M., Malli R., Graier W.F. (2016). Resveratrol Specifically Kills Cancer Cells by a Devastating Increase in the Ca^2+^; Coupling between the Greatly Tethered Endoplasmic Reticulum and Mitochondria. Cell. Physiol. Biochem..

[182] Yu H., Sun C., Gong Q., Feng D. (2021). Mitochondria-Associated Endoplasmic Reticulum Membranes in Breast Cancer. Front. Cell Dev. Biol..

[183] Selvaraj S., Mohan A., Narayanan S., Sethuraman S., Krishnan U.M. (2013). Dose-Dependent Interaction of trans-Resveratrol with Biomembranes: Effects on Antioxidant Property. J. Med. Chem..

[184] Karewicz A., Bielska D., Gzyl-Malcher B., Kepczynski M., Lach R., Nowakowska M. (2011). Interaction of curcumin with lipid monolayers and liposomal bilayers. Colloids Surf. B Biointerfaces.

[185] Sala de Oyanguren F.J., Rainey N.E., Moustapha A., Saric A., Sureau F., O’Connor J.-E., Petit P.X. (2020). Highlighting Curcumin-Induced Crosstalk between Autophagy and Apoptosis as Supported by Its Specific Subcellular Localization. Cells.

[186] Guo S., Lv L., Shen Y., Hu Z., He Q., Chen X. (2016). A nanoparticulate pre-chemosensitizer for efficacious chemotherapy of multidrug resistant breast cancer. Sci. Rep..

[187] Rashrash M., Schommer J.C., Brown L.M. (2017). Prevalence and Predictors of Herbal Medicine Use Among Adults in the United States. J. Patient Exp..

[188] Langin H., Lefebvre G., Tresch-Bruneel E., Deley M.-C.L., Marliot G., Sakji I., Vanseymortier M., Reich M., Lartigau E., Bonneterre J. (2018). Prevalence of herbal medicine (HM) use among breast cancer patients treated with chemotherapy, hormone therapy, or targeted therapy. J. Clin. Oncol..

[189] Morris K.T., Johnson N., Homer L., Walts D. (2000). A comparison of complementary therapy use between breast cancer patients and patients with other primary tumor sites. Am. J. Surg..

[190] Damery S., Gratus C., Grieve R., Warmington S., Jones J., Routledge P., Greenfield S., Dowswell G., Sherriff J., Wilson S. (2011). The use of herbal medicines by people with cancer: A cross-sectional survey. Br. J. Cancer.

[191] Orfi N.E., Boutayeb S., Rahou B.H., Aitouma A., Souadka A. (2021). Use of medicinal plants by cancer patients at the National Institute of Oncology, Rabat: A cross-sectional survey. Pan Afr. Med. J..

[192] Mao J.J., Palmer C.S., Healy K.E., Desai K., Amsterdam J. (2011). Complementary and alternative medicine use among cancer survivors: A population-based study. J. Cancer Surviv..

